# Foreword: the enhanced games—human enhancement, risk, and harm reduction in a post-doping era

**DOI:** 10.1186/s12954-026-01448-4

**Published:** 2026-04-17

**Authors:** Imran Khan, Luke Cox, Kyle T. Ganson, Timothy Piatkowski

**Affiliations:** 1 TransformNow, Birmingham, UK; 2 School of Sport and Exercise Science, Swansea University, Swansea, Wales UK; 3 Androgen and Hormone Research Centre, Swansea University, Swansea, Wales UK; 4 Centre for Health Services Research, Faculty of Health, Medicine, and Behavioural Sciences, University of Queensland, Brisbane, QLD Australia; 5 Factor-Inwentash Faculty of Social Work, University of Toronto, Toronto, ON Canada

## Introduction

The proposed Enhanced Games have emerged at a moment of increasing discussion and debate within sport, medicine, and public health regarding how human enhancement is conceptualised, governed, and regulated. By explicitly permitting the use of enhancement drugs (e.g., testosterone and human growth hormone) within an elite sporting context, the Enhanced Games challenge the prohibition-based anti-doping paradigm that has shaped international sport governance for decades. In doing so, the Enhanced Games draw renewed attention to long-standing but unresolved questions concerning bodily autonomy, acceptable risk, informed consent, commercialisation, and the role of harm reduction in contexts where enhancement practices already exist.

The Enhanced Games position themselves as rejecting concealment and prohibition in favour of transparency, regulation, and medical oversight. Proponents argue that acknowledging enhancement as an existing and enduring feature of elite sport may offer opportunities to manage risk more effectively than systems that formally prohibit use while struggling to eliminate it [1, 2]. Critics, conversely, raise concerns about escalation of harm, coercion, inequity, and the normalisation of pharmacologically intensified performance [3, 4]. These debates, while often polarised, underscore the need for careful empirical and conceptual examination rather than a priori endorsement or rejection.

From a harm reduction perspective, the Enhanced Games present an unusual and potentially informative case. Harm reduction has historically been applied to illicit drug use, sexual health, and other risk-laden behaviours where abstinence-only or punitive approaches have failed to prevent use, where many individuals do not wish to stop, and where such approaches have often exacerbated harm. The application of harm reduction principles to elite sport and performance enhancement, however, remains comparatively underdeveloped and contested [5, 6]. This Collection seeks to situate the Enhanced Games within this broader harm reduction literature, treating them as an object of scholarly inquiry rather than a policy position to be advocated or opposed.

## Enhancement as a social and biomedical practice

Human enhancement is neither a novel nor a marginal phenomenon. Across history, individuals and institutions have sought to extend physical capacity, endurance, cognition, and resilience through pharmacological, technological, surgical, nutritional, and training-based interventions [1]. In contemporary contexts, enhancement practices span clinical medicine, military performance, occupational productivity, aesthetic modification, and recreational fitness, blurring distinctions between medical use and enhancement [2, 7, 8].

In sport, pharmacological enhancement has been documented since at least the early twentieth century, predating the formation of formal anti-doping regimes [9]. The establishment of global prohibition systems, most notably through the World Anti-Doping Agency (WADA), has framed the use of enhancement drugs primarily as a rule violation rather than as a continuum of risk-bearing practices. These systems have also played an important role in reinforcing norms of fairness and maintaining spectator confidence in the legitimacy of sporting competition. While anti-doping governance has undoubtedly shaped sporting norms, enforcement has remained uneven, resource-intensive, and subject to ongoing technological and political contestation.

Research consistently demonstrates that enhancement practices persist even under prohibition, often adapting to detection thresholds, testing cycles, and regulatory blind spots [1]. Athletes may engage in complex dosing strategies, polypharmacy, and off-label use, frequently informed by informal networks rather than clinical guidance [10]. In this context, prohibition may function less as a deterrent than as a driver of concealment, delayed help and treatment seeking, and fragmented medical oversight [11].

The Enhanced Games explicitly challenge this model by proposing a sporting environment in which enhancement is acknowledged, transparent and regulated. Whether this reframing meaningfully alters risk trajectories, athlete behaviour, or health outcomes remains an empirical question to be answered/explored.

## Harm reduction and the governance of risk

Harm reduction is best understood not as an endorsement of risk, but as a pragmatic framework for reducing preventable harms associated with behaviours that persist despite regulation or prohibition [12]. Central to harm reduction are principles of realism, proportionality, participant autonomy, and engagement rather than punishment. Importantly, harm reduction does not assume that all harms can be eliminated, nor does it preclude broader regulatory or preventive strategies. Within drug policy, harm reduction interventions, such as supervised consumption sites, and drug checking, have demonstrated effectiveness in reducing morbidity and mortality [13]. These approaches have increasingly been recognised as compatible with, rather than opposed to, public health goals, especially when it comes to enhancement drugs [14].

The application of harm reduction in a competitive sporting setting raises distinct challenges. Unlike many public health contexts, elite sport is characterised by intense competition, commercial incentives, public visibility and buy-in, and institutional power asymmetries [15]. Risk is often valorised as a marker of commitment, resilience, and excellence. These dynamics complicate assumptions about voluntariness, informed consent, and the distribution of responsibility for harm. The Enhanced Games invoke harm reduction logic by proposing medical supervision, transparency of substance use, and monitoring of health indicators. In practice, this has been described as including pre-competition medical screening and health profiling, physician-supervised use of legally prescribed enhancement substances (e.g., testosterone), mandatory disclosure of substances used by athletes, and oversight from scientific or ethics advisory bodies. However, the presence of harm reduction language does not guarantee harm reduction outcomes. Where there is competition, high-stakes, and vast sums of prize money on offer to the most successful athletes, boundaries may become blurred and limits pushed, exposing athletes to vulnerability. Thus, critical examination is required to assess how such frameworks are operationalised, who controls them, and whose interests they ultimately serve.

## Responsibility, consent, and commercial contexts

One of the central questions raised by the Enhanced Games concerns responsibility for risk. In enhancement-permissive environments, responsibility may be distributed across athletes, teams, medical professionals, organisers, sponsors, and regulators. Determining where responsibility lies is complicated by power differentials, financial incentives, and cultural expectations surrounding performance and sacrifice [6]. Informed consent is often cited as a cornerstone of ethical enhancement models [16]. Yet consent in elite sport cannot be understood solely as an individual decision made in isolation. Athletes operate within structures that shape opportunity, risk, reward, and exclusion. Financial sponsorship, career precarity, national representation, and public scrutiny may all influence decision-making in ways that complicate straightforward notions of free choice [17].

The Enhanced Games provide an opportunity to examine how consent is constructed, documented, and revisited in enhancement-permissive contexts. Questions arise regarding the adequacy of information provided, the uncertainties associated with long-term health consequences, the role of independent medical advice, and mechanisms for withdrawal or refusal without penalty. Empirical research is needed to understand how athletes perceive and experience these processes, rather than assuming consent as a static or binary condition. Moreover, by explicitly expanding into the development and marketing of their supplement and testosterone brand, Enhanced positions sporting competition as a secondary objective, subordinated to its primary aim of capitalising on broader societal trends toward enhancement.

## Fairness, equity, and the meaning of competition

Fairness has long been central to anti-doping discourse, often framed as the primary ethical justification for prohibition [18]. Yet sociological and philosophical analyses have demonstrated that fairness in sport is neither absolute nor value-neutral [1, 2]. Competitive outcomes are shaped by unequal access to resources, training environments, medical care, and technological innovation. Enhancement practices exist along a continuum that includes altitude training, advanced nutrition, recovery technologies, surgical interventions, and pharmacological agents. Regulatory boundaries between “acceptable” and “unacceptable” enhancements are historically contingent, subjective, and frequently contested [1]. The Enhanced Games foreground these ambiguities by proposing explicit rules governing enhancement rather than attempting to eliminate it.

Whether such an approach produces a more or less equitable competitive environment is an open question. Some argue that transparency may reduce covert advantage and regulatory arbitrage; others contend that it may entrench inequalities by privileging those with access to superior medical support and resources. Comparative research examining outcomes under prohibition-based versus enhancement-permissive regimes is essential to advancing this debate beyond speculation.

## Public perception, spectatorship, and spillover effects

Beyond athletes themselves, the Enhanced Games raise questions about how enhancement is perceived by spectators and the broader public. Elite sport plays a powerful role in shaping cultural norms around bodies, performance, and success [2]. The visibility of enhancement practices may influence how non-athletes perceive the risk, legitimacy, and desirability of similar substances in fitness and lifestyle contexts.

Concerns have been raised regarding potential spillover effects, whereby promotional narratives surrounding enhancement may downplay risk or normalise intensive pharmacological use [3–6]. Such normalisation may also generate downstream clinical challenges, including the assessment and treatment of individuals who develop problematic patterns of enhancement drug use or experience related health complications. Conversely, transparency and discussion of risk may foster more informed decision-making among those already engaging in enhancement practices [19]. Empirical research is needed to understand how messaging, media coverage, and public discourse surrounding the Enhanced Games are interpreted and internalised.

Harm reduction scholarship has increasingly recognised the importance of examining not only direct participants, but also the broader social environments in which risk practices are embedded [20]. The Enhanced Games offer a novel case through which to explore these dynamics.

## Purpose of this collection

This Collection of *Harm Reduction Journal* is dedicated to examining the Enhanced Games as a complex social, biomedical, and regulatory phenomenon. *The Collection does not adopt a position for or against the Games*. Rather, it seeks to provide a rigorous scholarly forum in which claims, assumptions, and consequences can be empirically examined and critically analysed.

The Collection invites interdisciplinary contributions spanning public health, sociology, bioethics, medicine, law, sport science, and lived-living experience scholarship. Contributions may include empirical studies, comparative analyses, policy evaluations, theoretical explorations, and reflective commentaries. Lived-living experience perspectives are explicitly encouraged, recognising their critical contribution to understanding risk, decision-making, and harm reduction in practice.

Debates surrounding enhancement in sport are often polarised, moralised, and resistant to nuance. This Collection seeks to move beyond binary positions by foregrounding evidence, critical reflection, and interdisciplinary dialogue. The Enhanced Games present a unique context in which to examine how enhancement is governed when prohibition is not the organising principle.

Whether such models reduce harm, redistribute risk, or introduce new challenges cannot be assumed. These outcomes must be examined through careful research, ethical analysis, and engagement with those most directly affected. By assembling diverse scholarly perspectives, this Collection aims to contribute to a more informed and constructive conversation about enhancement, risk, and harm reduction in contemporary sport.
